# Management of an Oblique Cervical Root Fracture in an Immature Maxillary Central Incisor: A Case Report

**DOI:** 10.1155/crid/1273479

**Published:** 2026-06-04

**Authors:** Konstantinos Kodonas, Anastasia Fardi

**Affiliations:** ^1^ Department of Endodontology, School of Dentistry, Aristotle University of Thessaloniki, Thessaloniki, Greece, auth.gr; ^2^ Department of Dentoalveolar Surgery, Implantology and Oral Radiology, School of Dentistry, Aristotle University of Thessaloniki, Thessaloniki, Greece, auth.gr

**Keywords:** calcium silicate cement, case report, dental trauma, immature permanent tooth, oblique cervical root fracture, vital pulp therapy

## Abstract

Traumatic dental injuries in immature permanent teeth present significant diagnostic and therapeutic challenges, particularly when associated with root fractures. A 7‐year‐old boy presented with trauma to the anterior maxilla after a fall on the pavement. Right central incisor (#11), previously unerupted due to premature extraction of the primary predecessor, erupted rapidly within 2 weeks post‐trauma, likely facilitated by dental trauma. Initial radiographic examination revealed an immature root with an open apex (Cvek Stage 1) and an oblique root fracture extending from the cervical region toward the middle–apical third. No other clinical signs were present. The patient remained asymptomatic throughout the observation period, reporting no pain or discomfort at any time. Cone beam computed tomography (CBCT) obtained 3 months later confirmed the root fracture and demonstrated dentin bridge formation, isolating the coronal pulp associated with the root fracture from the unaffected pulp. A sinus tract developed 6 months post‐trauma, but definitive treatment was delayed because of insufficient clinical crown for access. A month later, sufficient eruption allowed access cavity preparation. Under high magnification, a thick, continuous tertiary dentin bridge was observed overlying the pulp, with a localized pulpal exposure at the junction with the distal dentinal wall; bleeding confirmed pulpal vitality. The coronal pulp space above the bridge was gently irrigated with 1% NaOCl and 17% EDTA without mechanical instrumentation and subsequently sealed with a calcium silicate‐based cement up to 2 mm below the cementoenamel junction. Clinical and radiographic follow‐up for up to 2 years showed continued root development, dentin bridge formation, canal narrowing, and absence of symptoms. Conservative vital pulp therapy may be a viable treatment option in selected cases of root fractures in immature permanent teeth when pulp vitality is preserved.

## 1. Introduction

Traumatic root fractures in immature permanent teeth are uncommon but present distinct clinical and biological challenges due to the incomplete root development and high susceptibility to pulpal complications. Root fractures account for approximately 0.5%–7% of injuries to permanent teeth and most frequently affect the maxillary anterior region of children following direct trauma [1, 2]. These injuries involve the dentin, cementum, pulp, and periodontal tissues, with the middle third of the root most commonly affected [3, 4]. In children (7–9 years), incomplete root formation and open apices increase the risk of adverse outcomes [5, 6]. The degree of the coronal fragment displacement is a key prognostic factor, as it may compromise the neurovascular supply and pulpal survival and healing [7]. Accurate diagnosis, relying on clinical and advanced radiographic evaluation, is essential for treatment planning [8, 9].

Healing outcomes vary depending on the type and severity of the fracture and may include calcified tissue repair, connective tissue interposition, mixed hard and soft‐tissue healing, or nonhealing associated with inflammatory granulation tissue and infection [7, 10]. When properly managed, root fractures in immature permanent teeth generally have a favorable prognosis, depending on the fracture′s location and direction, the degree of displacement, pulpal vitality, and the biological healing response [11].

Tertiary dentinogenesis represents an inherent defense and repair response of the dentine–pulp complex, initiated by noxious stimuli such as trauma, and mediated by surviving odontoblasts or newly differentiated odontoblast‐like cells that deposit reactionary or reparative dentin, respectively, to protect the pulp [12, 13]. The application of bioceramic materials optimizes this repair by providing a bioactive, hermetic seal that induces hydroxyapatite formation and stimulates periradicular tissue regeneration. Bioceramic materials, particularly mineral trioxide aggregate (MTA), can stimulate tertiary dentin formation by releasing calcium ions and creating an alkaline environment that promotes growth factor release, stem cell recruitment, and odontoblastic differentiation at the pulp–dentin interface, thereby enhancing the natural regenerative capacity of the dentine–pulp complex [14, 15]. Owing to their excellent sealing ability, biocompatibility, antibacterial properties, and capacity to stimulate mineralized barrier formation, bioceramic materials such as MTA are widely employed in the management of root fractures, with favorable long‐term outcomes reported in the literature [16–20].

The purpose of this report is to present the clinical management and 2‐year follow‐up of an oblique cervical root fracture in a maxillary permanent right central incisor (tooth #11) of a 7‐year‐old boy who sustained trauma before complete tooth eruption, highlighting diagnostic considerations and treatment outcomes in an immature permanent tooth. The affected tooth presented with an open apex, classified as Cvek Stage I, indicating an immature stage of root development [21].

Although root fractures are well‐documented in the permanent dentition, their occurrence in unerupted teeth is extremely rare. To the best of our knowledge, this is a rare report describing this particular sequence of events and a favorable outcome in an immature tooth with a cervical root fracture and delayed definitive management. This case highlights the diagnostic challenges, distinct biological response, and treatment considerations that differ markedly from typical root fractures in erupted teeth, adding valuable insight to the limited literature on complex pediatric dental trauma.

## 2. Case Presentation

A 7‐year‐old Greek boy was referred after a fall on the sidewalk, during which he struck a parking post (bollard), resulting in trauma to the anterior maxilla. The patient was clinically healthy, with no significant medical history or contraindicating systemic conditions, and presented with an otherwise unremarkable dental history.

At the time of injury, the child was evaluated at a primary regional healthcare unit, where management focused on the soft‐tissue injury, and no periapical or occlusal radiographs were obtained. At the initial visit (T0), clinical examination revealed a soft‐tissue abrasion with mild bleeding and localized swelling overlying the impacted right central incisor (tooth #11), which had not yet erupted at the time of the trauma due to the premature extraction of its primary predecessor. No deep lacerations, foreign bodies, mucosal defects, or alveolar fractures were identified. Percussion and palpation of the adjacent erupted teeth were painless, with no tenderness indicative of alveolar involvement.

Two weeks post‐trauma, the patient was referred for an endodontic evaluation of the maxillary anterior teeth. At that time, part of the crown of tooth #11 was visible to the oral cavity due to the ongoing eruption process. Written informed consent was obtained from the patient′s legal guardian for treatment.

Periapical radiography revealed an immature open apex (Cvek Stage 1) and a root fracture line extending obliquely from the cervical region toward the middle–apical third (Figure 1a). Periapical tissues and adjacent teeth appeared normal. The ongoing eruption of the tooth limited clinical evaluation. Sensibility testing was not performed because only a small portion of the tooth crown erupted and was clinically manageable (Figure 1b). Percussion and palpation were painless; no mobility or swelling was noted, and adjacent teeth remained asymptomatic (Table 1). Cone beam computed tomography (CBCT), performed 3 months later, confirmed the oblique root fracture and revealed tertiary dentin formation isolating the pulp in contact with the root fracture line from the unaffected pulp (Figure 1c–e). For the subsequent period, the patient presented no symptoms, the eruption process continued normally, and the patient was monitored every 15 days (Figure 2a,b). Six months post‐trauma, a sinus tract developed; no pain was reported. A radiopaque gutta‐percha point was gently inserted into the sinus tract until slight resistance was felt, followed by radiographic imaging to trace its course. (Figure 2c,d). Subsequent intervention was not feasible due to the limited extent of the erupted clinical crown, which precluded adequate isolation and safe access for treatment. This limitation also justified the decision to delay definitive therapy following the development of a sinus tract until sufficient eruption allowed appropriate access.

**Figure 1:**
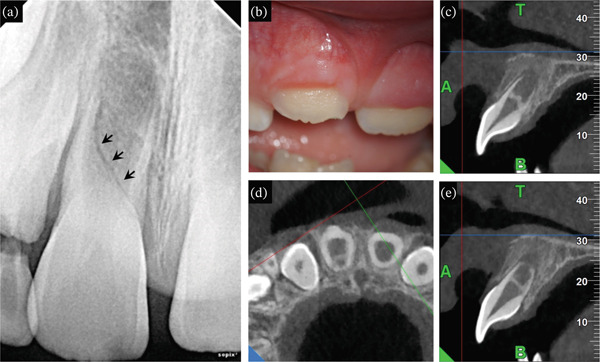
(a) Diagnostic imaging of the traumatized immature maxillary central incisor (#11). Periapical radiograph showing Cvek Stage 1 root development and an oblique fracture line extending from the cervical region toward the middle–apical third (arrows). (b) Clinical view during eruption, showing tooth #11 15 days post‐trauma. (c–e) CBCT scans obtained 3 months post‐trauma, confirming the oblique fracture and demonstrating tertiary dentin formation, isolating the root pulp from the fracture line. (d) axial (c,e) sagittal sections of #11.

**Table 1:** Chronological timeline of clinical findings, treatment, and diagnostic test results.

Time point	Clinical Findings	Treatment/action	Diagnostic sensibility tests (percussion, palpation findings, and results from control teeth). Patient‐reported symptoms
Initial Trauma/T0 (Baseline)	Acute trauma to anterior maxillary region	Initial clinical examination without imaging	**Soft-tissue**: abrasion with mild bleeding and localized swelling over impacted tooth #11 **Palpation**: Painless **Percussion**: Painless **Occlusal interference**: None **Sensibility testing**: Not applicable **Mobility testing**: Not applicable **Patient-reported symptoms**: None
Two weeks post‐trauma	Almost unerupted maxillary incisor with oblique root fracture	Clinical and radiographic examination. (Figure 1a,b)	**Periapical tissues/control teeth**: Normal **Sensibility testing**: Not applicable because ongoing tooth eruption allowed evaluation of only a small portion of the crown **Palpation**: Normal **Percussion**: Painless **Mobility/swelling**: None **Patient-reported symptoms**: None
Month 3 post‐trauma	Partial eruption of the maxillary incisor	Clinical examination and CBCT (Figure 1c–e)	**Sensibility testing**: Not applicable **CBCT**: Calcified barrier formation indicative of tertiary dentin isolated the pulp in contact with the fracture line. **Patient-reported symptoms**: None
Month 6 post‐trauma	Symptom onset, sinus tract development	Treatment was delayed/deferred because the limited erupted portion of the crown restricted palatal access for intervention. (Figure 2c,d)	**Periapical tissues/control teeth**: Normal **Mobility/swelling**: None **Palpation**: Normal **Percussion**: Painless **Patient-reported symptoms:** None
Month 7 post‐trauma	Sinus tract persisted, Calcified barrier formation	Initiation of treatment with Bio‐C Repair (calcium silicate‐based bioceramic material). (Figure 3a–c)	**Periapical tissues/control teeth**: Normal **Sensibility testing of #11:** Negative **Sensibility testing of control:** Normal **Mobility/swelling**: None **Palpation**: Normal **Percussion**: Painless **Patient-reported symptoms**: None
Month 8 post‐trauma	Complete eruption	Final restoration and follow‐up. (Figure 4a)	**Periapical tissues/control teeth**: Normal **Mobility/swelling**: None **Patient-reported symptoms:** None **Palpation**: Normal **Percussion**: Painless
Long‐term Follow‐up 6 months, 1 and 2 years	Normal periodontal healing	Successful outcome confirmed by clinical and radiographic examination. (Figure 4b–d)	Continued root development with apical maturation and closure, progressive thickening of the dentinal walls, and increased root length, almost matching the maturation pattern of the adjacent tooth

**Figure 2:**
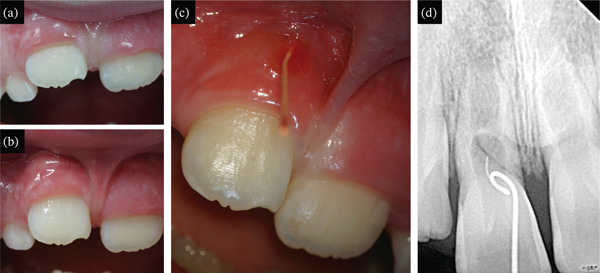
(a) Post‐trauma clinical and radiographic views of the immature maxillary incisor (#11). Clinical view at 4 months. (b) Clinical view at 5 months. (c) Clinical view at 6 months following trauma, with gutta‐percha placed in the sinus tract. (d) Corresponding periapical radiograph.

Seven months following trauma (Figure 3a), the eruption allowed access for cavity preparation. At that stage, a combination of clinical and intraoral findings, including the absence of spontaneous pain, lack of tenderness to percussion and palpation, and the absence of swelling, inferred probable pulp vitality. Sensibility testing was not considered reliable due to incomplete root formation and limited coronal structure (Table 1). After entering the pulp chamber and under high magnification with the operating microscope, the tertiary dentin (dentinal bridge) overlying the pulp was clearly visualized (Figure 3b,c). The root fracture line was also clearly visible, extending obliquely from the cervical buccal region into the coronal and middle thirds of the root. The dentin bridge appeared smooth, thick, and structurally continuous, with no detectable defects or gaps along its surface. However, a localized area of pulp exposure was located at the junction between the bridge and the distal longitudinal dentinal wall (Figure 3c). The presence of bleeding at this site confirmed pulpal vitality. This clinical presentation is consistent with the formation of a mature tertiary dentinal bridge that has provided substantial coverage of the underlying pulp tissue, while still allowing a limited, localized exposure through which pulpal vitality could be objectively verified.

**Figure 3:**
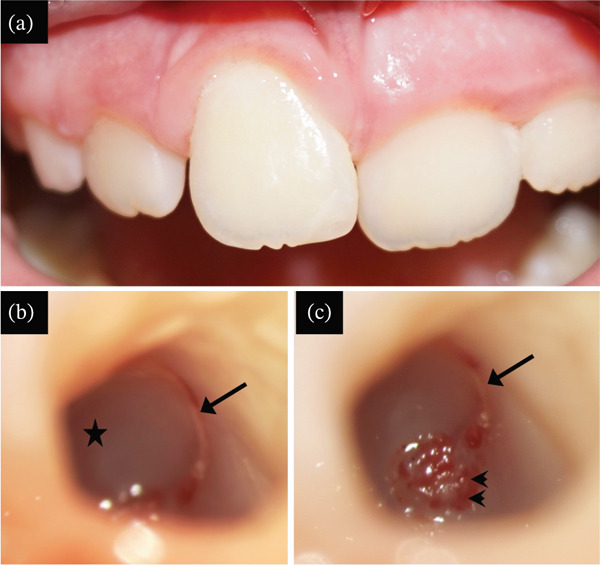
(a) Clinical and microscopic findings 7 months post‐trauma. Clinical view of tooth #11. (b,c) High‐magnification clinical views showing a dentinal bridge over the root pulp (asterisk), the fracture line extending obliquely from the cervical buccal region into the coronal and middle thirds of the root (arrow), and a localized area of pulp exposure between the dentin bridge and the distal longitudinal dentinal wall (arrowhead).

The portion of the pulp space coronal to the dentinal bridge appeared largely devoid of vital pulp tissue, with only sparse remnants of soft tissue adherent to the canal walls and no clinically detectable exudate suggestive of ongoing inflammation or infection. The area was gently irrigated with 15 mL of 1% NaOCl, delivered with a side‐vented needle positioned 1 mm short of the dentinal bridge, to achieve chemical disinfection [22]. Irrigation was continued until the solution was clear and no visible tissue remnants remained. Subsequent irrigation with 5 mL of 17% EDTA followed by a final irrigation of 5 mL of NaOCl was performed to remove the smear layer, improve disinfection of the dentin surface, and promote optimal interaction between the dentin and the capping material [23]. No mechanical instrumentation was performed to preserve the residual dentin structure and reduce the risk of perforation. After completing the irrigation protocol and confirming the absence of bleeding, the coronal pulp space above the dentinal bridge was considered adequately disinfected and subsequently dried using aspiration and sterilized paper points. The vital tissue and pulp space above the dentin bridge were capped with Bio‐C Repair (Angelus, Londrina, PR, Brazil). This calcium silicate‐based cement was used to fill all available space from the dentin bridge to 2 mm below the CEJ (Figure 4a). Although MTA remains the gold standard for pulpotomy due to its well‐established biocompatibility and clinical success, newer hydraulic calcium silicate‐based bioceramics such as Bio‐C Repair have been developed to address limitations in handling and setting time, while demonstrating comparable clinical performance and sealing ability, particularly in vital pulp therapy of immature permanent teeth following trauma [24, 25]. Clinical and radiographic follow‐up evaluations at 6 months, 1 and 2 years demonstrated continued root development with apical maturation and closure, progressive thickening of the dentinal walls, and increased root length, almost matching the maturation pattern of the distally located tooth #21 (Figure 4b,c,d). Radiographically, a progressively thicker dentin bridge was evident, accompanied by gradual narrowing of the root canal space and a well‐defined apical outline. Throughout the observation period, the patient remained asymptomatic, with no evidence of pain, sinus tract, swelling, pathologic mobility, or periodontal symptoms associated with the tooth. The tooth has remained functional, although a mild overjet persists and is planned to be addressed with orthodontic treatment.

**Figure 4:**
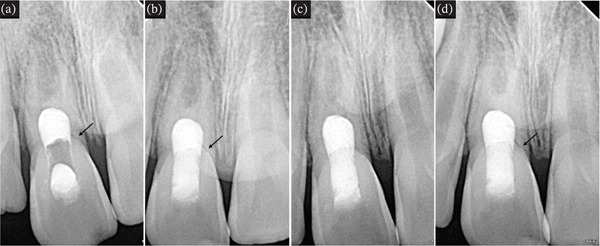
(a) Post‐treatment radiograph of tooth #11 showing calcium silicate‐based cement filling the space from the dentin bridge to a level 2 mm below the CEJ. (b–d) Radiographic follow‐ups at 6 months (b), 1 year (c), and 2 years (d) demonstrating continued root development and apical maturation. The arrow indicates the fracture line at the cervical portion of the root near the CEJ.

## 3. Discussion

This case illustrates the capacity of immature pulp to induce a reparative response following trauma, even in the presence of an oblique cervical root fracture, with CBCT imaging proving essential for diagnosis and treatment planning. In the present report, conservative vital pulp therapy preserved root pulp vitality and allowed continued root development, emphasizing the value of a biologically oriented treatment strategy in immature traumatized incisors. The patient reported no symptoms and remained comfortable during the treatment period, expressing satisfaction with the overall outcome.

The clinical and radiographic findings in this case—formation of a thick, continuous dentin bridge, preservation of apical pulp vitality, and continued root development—underscore the remarkable self‐reparative potential of the immature dentin–pulp complex. They are consistent with experimental and clinical evidence supporting successful vital pulp therapy in immature teeth [25–29] and with the view that the dentin–pulp complex is a dynamic tissue capable of reparative reactions, mediated by a highly vascular, cell‐rich pulp containing undifferentiated mesenchymal cells [30, 31]. The presence of a mature dentin bridge in association with an oblique cervical root fracture is particularly noteworthy. Root fractures are traditionally viewed as unfavorable prognostic indicators because they may act as pathways for bacterial ingress and fluid movement, potentially leading to pulpal inflammation and necrosis [32]. In this case, however, the pulp demonstrated sufficient vitality and organization to form tertiary dentin strategically below the root fracture trajectory, effectively sealing the communication with the unaffected root pulp. This is consistent with the concept that, under specific conditions, that is, when bacterial contamination is limited, and pulpal inflammation remains controlled, the dentin–pulp complex can produce adequate tertiary dentin to isolate the injured zone and restore an effective barrier [12, 28]. Compared with previous reports that often describe compromised prognosis in fractured roots [32], the present case suggests that in immature teeth with controlled bacterial challenge, tertiary dentinogenesis may partially compensate for the structural defect and support a more favorable outcome.

A noteworthy aspect of this case is the timing and nature of the first clinical symptom. It appeared only when the fracture line came into functional communication with the septic environment of the periodontal ligament space, as evidenced by the later development of a sinus tract. During the earlier eruptive phase, the fracture remained insulated from the oral and periodontal microbiota. The absence of contamination appears to have allowed the dentin–pulp complex to develop an organized tertiary dentin response without triggering severe inflammatory breakdown, which is consistent with experimental data showing that the quality and intensity of pulpal inflammation are strongly correlated with bacterial presence and load [33–35]. This sequence supports the concept that bacteria, rather than mechanical trauma alone, are the primary causes of irreversible/severe pulpal pathology, and that in their absence, even structurally compromised roots in immature teeth can maintain vitality and sustain reactionary and/or reparative dentinogenesis through a mild or moderate inflammatory response [31, 36].

Following tooth eruption, the fracture line communicated with the periodontal ligament space, the oral environment, and, consequently, with a polymicrobial biofilm. Consequently, a localized infectious pathway developed and ultimately presented clinically as a sinus tract, even though the apical pulp remained vital. The development of a sinus tract in the presence of presumed pulp vitality may appear paradoxical and therefore warrants clarification. In the present case, diagnostic reasoning was based on a combination of clinical and radiographic findings. Throughout the follow‐up period, the tooth remained asymptomatic, with no tenderness to percussion or palpation, no pathologic mobility, and no radiographic signs of apical pathology. These observations suggest that the sinus tract resulted from a localized breakdown associated with infection along the cervical fracture pathway, communicating with the periodontal ligament space, rather than from generalized necrosis of the entire root canal system. This concept is consistent with the view that bacterial ingress, rather than mechanical trauma alone, is the principal determinant of severe pulpal breakdown [37]. Clinically, this highlights the importance of early identification and control of bacterial pathways in root fractures of immature teeth to preserve pulp vitality.

Despite the presence of a sinus tract, definitive treatment was deliberately postponed because the limited erupted clinical crown did not allow for reliable rubber dam isolation or safe palatal access preparation. Attempting endodontic intervention under these conditions would have significantly increased the risks of contamination, iatrogenic damage, and compromised long‐term prognosis. The tooth remained asymptomatic, with no tenderness to percussion or palpation and normal radiographic appearance of the periapical tissues, suggesting that the apical pulp was still vital and that immediate intervention was not mandatory. Therefore, a risk–benefit assessment favored close clinical and radiographic monitoring until sufficient eruption permitted controlled access, isolation, and minimally invasive treatment.

The 15‐day follow‐up interval was established to closely monitor the hemodynamic and eruptive dynamics of the immature incisor. Given the importance of early pulp necrosis detection and the impact of loading forces during active eruption, frequent clinical assessments ensured that any signs of pulpal complications or unfavorable fragment displacement during eruption were identified and addressed promptly. This approach is consistent with the need for individualized protocols in complex traumatic cases [7, 38]. Nonetheless, according to the International Association of Dental Traumatology (IADT) guidelines, clinicians should customize follow‐up schedules based on the complexity and severity of the traumatic injury, as well as the developmental stage of the root [39].

The choice of a low‐concentration sodium hypochlorite solution (1% NaOCl) was based on the need to balance effective chemical disinfection with minimizing the risk of compromising residual vital pulp. The subsequent use of 17% EDTA was intended to remove the smear layer, facilitating deeper penetration of the disinfecting solution and optimizing the interaction between dentin and the calcium silicate‐based material. This irrigation protocol, performed without mechanical instrumentation, sought to maximize intracanal disinfection and improve the quality of the dentin surface for sealing, while minimizing the risk of further damage to the residual vital pulp of the immature root.

A further consideration in this case is the limited diagnostic value of sensibility testing. Due to incomplete root development, a condition under which sensibility tests frequently yield false‐negative or inconsistent responses, and the restricted coronal structure of partially impacted tooth #11, conventional sensibility tests could not be applied reliably [40]. Consequently, the diagnosis of pulp vitality in the present case relied primarily on subsequent clinical and radiographic findings, including the absence of clinical symptoms and the radiographic evidence of continued root development. This should be considered a diagnostic limitation of the present report.

Another limitation of this case was the absence of radiographic assessment at the initial trauma visit, which was not in accordance with current trauma guidelines. Early imaging might have enabled earlier diagnosis and more structured follow‐up. This case also reflects real‐world practice in nonspecialist or resource‐limited settings, where omission of imaging can significantly influence the diagnostic pathway. Furthermore, CBCT was obtained 3 months after the injury rather than at the time of trauma to balance the need for three‐dimensional diagnostic information with the obligation to minimize radiation exposure. At the initial visit, periapical radiography, together with the limited clinical access due to incomplete eruption, provided sufficient information to establish a provisional diagnosis and to initiate close clinical monitoring. In addition, parental agreement for CBCT was not granted at that stage. Only after 3 months did further eruption of tooth #11 allow for more detailed visualization of the fracture line and its relationship to the root and surrounding structures, as well as the developing dentinal bridge, a fact that was critical for treatment planning. Tertiary dentin is classically categorized as reactionary dentin, produced by surviving primary odontoblasts in response to mild stimuli, or reparative dentin, laid down by newly differentiated odontoblast‐like cells after localized cell death induced by stimuli of moderate intensity [26, 27]. Reactionary dentin is typically continuous with primary and secondary dentin and often exhibits a tubular pattern, whereas reparative dentin is usually more irregular and heterogeneous or atubular, showing no continuity with primary dentin [27, 28]. In the present case, CBCT and microscopic evaluation suggested tertiary dentin deposition over the fracture line at the interface with the remaining vital root pulp. The dentinal bridge appeared radiographically thick and continuous and, under magnification, smooth and structurally coherent [13, 29]. Nonetheless, the absence of histologic confirmation precludes definitive conclusions regarding the nature and quality of the reparative hard tissue.

The decision to avoid more invasive treatment was based on evidence of preserved pulp vitality, continued root development, and dentin bridge formation, indicating ongoing healing. Accordingly, vital pulp therapy was selected, as such procedures are generally preferred in immature teeth when pulp vitality can be maintained. In clinical endodontics, magnification enhances visualization of fine anatomical details and defects, thereby improving diagnostic accuracy and the precision of operative procedures [41]. In addition, three‐dimensional imaging with cone beam CT has proven critical for assessing both the extent of structural damage and the spatial distribution of reparative hard tissue formation, providing information that cannot be obtained from conventional two‐dimensional radiographs [42–44]. Following careful assessment, when vitality is present, vital pulp therapy using bioactive calcium silicate‐based materials can effectively support the pulp′s healing capacity [45, 46]. Calcium silicate cements such as MTA and related bioceramic materials have been shown to support odontoblastic differentiation, stimulate tertiary dentinogenesis, and provide a tight coronal seal, thus favoring long‐term maintenance of pulp vitality [15, 16, 46]. Bio‐C Repair, a premixed calcium silicate‐based material, has been shown to exhibit good biocompatibility and promineralization potential on human dental pulp stem cells, supporting its regenerative capacity in vital pulp therapy. These findings, together with its favorable physicochemical properties, justify its use in direct pulp capping procedures aimed at preserving pulp vitality [47, 48].

Overall, this case reinforces the concept that the dentin–pulp complex acts as a self‐repairing biological system. When adequately protected from persistent bacterial contamination, the pulp can stimulate the formation of tertiary dentin, supporting root maturation and long‐term tooth function. These observations are in line with contemporary biologically based endodontic concepts that prioritize preservation of pulp vitality and stimulation of the tooth′s own reparative potential whenever feasible [31, 45]. At the same time, the single‐case nature of this report, the absence of histologic confirmation, and the limited follow‐up period constrain the generalizability of the findings and underline the need for larger clinical studies to better define indications and prognosis for this approach.

## 4. Conclusion

Vital pulp therapy utilizing a calcium silicate‐based material may be considered an appropriate treatment option for immature teeth exhibiting oblique cervical root fractures, if pulp vitality is maintained. However, this favorable outcome should be interpreted with caution and should not be generalized to all types of root fractures. Treatment decisions and prognosis should be guided by the specific characteristics of the fracture pattern, as well as clinical findings and the status of pulp vitality.

## Author Contributions

**Konstantinos Kodonas:** conceptualization, methodology, validation, investigation, resources, writing – review and editing, supervision, project administration. **Anastasia Fardi:** software, validation, formal analysis, data curation, writing – original draft, visualization.

## Funding

The publication of this article in OA mode was financially supported by HEAL‐Link.

## Disclosure

All authors have read and approved the final version of the manuscript. Konstantinos Kodonas had full access to all of the data in this study and takes complete responsibility for the integrity of the data and the accuracy of the data analysis.

## Ethics Statement

Ethical approval was not required for this study because it is a single anonymized case report. Written informed consent for publication of all clinical information and images was obtained from the patient′s legal guardian(s).

## Conflicts of Interest

The authors declare no conflicts of interest.

## Data Availability

The data that support the findings of this study are available on request from the corresponding author. The data are not publicly available due to privacy or ethical restrictions.
